# Effects of Magnesium Oxide (MgO) Shapes on In Vitro and In Vivo Degradation Behaviors of PLA/MgO Composites in Long Term

**DOI:** 10.3390/polym12051074

**Published:** 2020-05-08

**Authors:** Yun Zhao, Hui Liang, Shiqiang Zhang, Shengwei Qu, Yue Jiang, Minfang Chen

**Affiliations:** 1 School of Materials Science and Engineering, Tianjin University of Technology, Tianjin 300384, China; yun_zhaotju@163.com (Y.Z.); huiliang2014@126.com (H.L.); 18522141052@163.com (S.Z.); qsw98810@163.com (S.Q.); jiang666yue@163.com (Y.J.); 2 Key Laboratory of Display Materials and Photoelectric Device (Ministry of Education), Tianjin 300384, China; 3 National Demonstration Center for Experimental Function Materials Education, Tianjin University of Technology, Tianjin 300384, China

**Keywords:** biopolymers composites, MgO nanoparticles, MgO whiskers, PLA, in vitro degradation, in vivo degradation

## Abstract

Biodegradable devices for medical applications should be with an appropriate degradation rate for satisfying the various requirements of bone healing. In this study, composite materials of polylactic acid (PLA)/stearic acid-modified magnesium oxide (MgO) with a 1 wt% were prepared through blending extrusion, and the effects of the MgO shapes on the composites’ properties in in vitro and in vivo degradation were investigated. The results showed that the long-term degradation behaviors of the composite samples depended significantly on the filler shape. The degradation of the composites is accelerated by the increase in the water uptake rate of the PLA matrix and the composite containing the MgO nanoparticles was influenced more severely by the enhanced hydrophilicity. Furthermore, the pH value of the phosphate buffer solution (PBS) was obviously regulated by the dissolution of MgO through the neutralization of the acidic product of the PLA degradation. In addition, the improvement of the in vivo degrading process of the composite illustrated that the PLA/MgO materials can effectively regulate the degradation of the PLA matrix as well as raise its bioactivity, indicating the composites for utilization as a biomedical material matching the different requirements for bone-related repair.

## 1. Introduction

The requirement for orthopedic implants in clinical medicine has been paid more attention by researchers in order to develop new materials to help patients avoiding the pain of secondary surgery, improve the recovery rate and reduce costs. Recently, polylactic acid (PLA) has been given more scientific research in the biomedical field, especially for bone repair [1,2,3,4], since it has the advantages of good biocompatibility, processability, biodegradability and bioabsorbability. However, some disadvantages have limited its widening applications [5,6,7,8]. For example, the mechanical properties of PLA cannot satisfy the requirements of load-bearing devices for bone fixation [5], and its poor bioactivity would significantly hinder the penetration and absorption of cells on the material surface [8]. In particular, predominant problems in its degradation have occurred. The pH variation in the degradation can accelerate the initial degradation rate of the implant and also increase the risk of adverse tissue reactions, such as the inflammatory response [9], which are not beneficial to the viability of bone cells, biodegradation properties and the requirements of bone formation.

In order to solve these problems, researchers have improved the performance of PLA by using inorganic fillers with a good biological activity and bone-similar ingredients [10,11,12]. Magnesium oxide (MgO), another kind of inorganic filler, is with good biocompatibility and non-toxic biological activity, and become a research hotspot of polymer modification [13,14,15]. It can release the Mg^2+^ ions when dissolving, which is favorable for a variety of enzymes conducive to the activation and synthesis of proteins. Moreover, its unique biological activity plays an important role in cell viability and as the antibacterial agent, to improve the survival rate of cells as well as the biological activity of composites [7,16]. Additionally, MgO also perform as an alkaline degradable material with excellent biological characteristics for the tissue engineering of regenerated bone tissue [16]. It is worth mentioning that besides the release of magnesium ions in the dissolving process, MgO, including the nanoparticles and whiskers, can impact on increasing the mechanical properties of the polymer matrix [13,17] and this is very different from the pure Mg particles as a filler in the PLA matrix [18]. It shows that since the Mg materials can produce the hydrogen during the composite degradation, it is easily apt to form the interface defects between the matrix and fillers, and meanwhile, there is no surface modification for the Mg particles prior to the preparation of the composite, which also weakens the interface bonding. In previous studies [19], it is found that the PLA/MgO composite film fabricated by the solution casting method can significantly improve the mechanical properties and biological activities of the PLA matrix, and its alkalinity has a neutralizing effect on regulating the pH of short-term degradation solutions.

It has been reported that the shape of fillers plays a crucial role in the mechanical properties and crystalline behaviors of composites. As to the materials of the PLA matrix, the whiskers usually perform the more enhancing capacity for the mechanical properties of PLA, compared with the nanoparticles [7,19]. Moreover, the difference in filler shape also affects the degrading behavior of the PLA composite during a short-time period because the dissolving rates of fillers are diverse, and this can cause the different variation in the interface bonding [18]. Luo et al. studied the degradation properties of MgO whisker/PLA composites in vitro, focusing on the long-term degradation, and since, their work has proved its better mechanical property. Nonetheless, it is necessary not only to focus on the initial property, but also to understand and investigate the long-term degradability and biocompatibility of such composites, as implants for bone repair, especially the filler shape effects on bone cells and tissues. It is worth noting that the change in the hydrophilicity and crystallization of the polymeric matrix has also been improved and influenced by the addition of fillers as well as its shape. The increasing hydrophilicity can cause the variation in the water intake in materials, and the change in the crystallization is beneficial to the growth of ordered crystal structures by affecting the degradation process of the matrix, such as the hydrolysis process of PLA, as the chain and segment are more easily apt to move in the degradation [20,21]. However, few studies have focused on the effects of the MgO shape on the degrading property of the PLA/MgO composite, and there is also no report on the in vivo degradation process of the PLA/MgO composite which provides the direct performance of its degrading property and biocompatibility to bone tissue.

In this work, MgO nanoparticles and whiskers are modified with stearic acid to obtain chemical binding through their interactions [22], as demonstrated in our previous work [17]. The PLA/MgO composites soaked in a phosphate buffer solution (PBS) under long-term degradation were studied, and particularly, the influence of the filler shape on the degradation behavior of the composites was analyzed. The in vivo degradation behavior of the composites was also evaluated.

## 2. Materials and Methods

### 2.1. Materials

PLA 2002D (NatureWorks, USA) was dried at 40 °C for 72 h before use and all other agents used were of analytical grade and did not require further treatment.

### 2.2. Preparations and Modifications of pMgO and wMgO

The fillers of the MgO nanoparticles (pMgO) and MgO whiskers (wMgO) were prepared in the laboratory [17,23]. Briefly, MgCl_2_·6H_2_O (76.24 g, CAS: 7791-18-6) was dissolved in 250 mL deionized water, and 1 mL cetyl trimethyl ammonium bromide (CTAB) (1.0 wt%, CAS: 57-09-0) was added dropwise. C_2_H_2_O_4_·2H_2_O (23.64 g, CAS: 6153-56-6) was added to this solution and the mixture was allowed to react for 20 min. The suspension was collected and centrifuged at 7000 rpm for 10 min. The pMgO precipitate was dried for 1 h in a vacuum oven (85 °C) and then sintered in a muffle furnace for 5 h at 600 °C.

The 100 mL solution of Na_2_CO_3_ (0.6 mol/L, CAS: 497-19-8) was added dropwise into an equal volume solution of MgCl_2_ (0.6 mol/L, CAS: 7791-18-6) and stirred for 20 min. The mixture was aged at room temperature for 10 h and then filtered, washed, and dried at 80 °C for 3–4 h. The precursor was calcined at 750 °C for 4 h with a heating rate of 5 °C/min. Then, the resultant wMgO was obtained.

Mixtures of 0.5 g of MgO (nanoparticles or whiskers) and 50 mL of ethanol were placed in three-necked flasks, which were subjected to ultrasonic treatment in a bath to achieve a full dispersion of the mixtures. The mixtures were then heated to 45 °C under reflux condensation. Stearic acid (0.005 g, CAS: 57-11-4) was dissolved into 20 mL of ethanol, added dropwise into each MgO suspension, and allowed to react for 1 h in reflux condensation conditions. The mixtures were then centrifuged, washed, and dried. The resultant products were denoted as SpMgO or SwMgO. The morphologies of pMgO, wMgO, SpMgO and SwMgO are shown in the Supplementary Materials.

### 2.3. Preparation of PLA/pMgO and PLA/wMgO Composites

The PLA/pMgO and PLA/wMgO composites were manufactured by a micro twin screw extruder (Wuhan Ruiming Test Equipment, Ltd., China) in advance, with a screw speed of 40 rpm and the temperature profile varied from 165 °C at the feeding zone to 190 °C at the die. The SpMgO/SwMgO was dry-mixed with PLA and the mixtures were dried under a vacuum at 50 °C for 48 h prior to the preparing process for the PLA and composites, then followed by the pelletizing step. Detailed information on the prepared materials is presented in Table 1.

The extruded pellets were dried at 50 °C for 24 h before extrusion again by the same machine conditions to obtain the composites’ wire (φ2 mm, controlled by the extrusion machine head) and then prepared the specimens’ rods (φ2 × 10 mm) directly. These extrusion-molded specimens were used for the in vitro degrading experiments.

### 2.4. In Vitro Degradation in PBS

In vitro degradation of the pure PLA and composite rods were carried out by immersing them in 4 mL of a phosphate buffer solution (PBS) at 37 °C for 3, 4, 5, 6, 8 and 12 months. The specimens of each material were removed from the PBS at the end of these periods. After rinsing with deionized water and removing the surface water using filter paper, the samples were weighed and then dried in a vacuum at 40 °C to a constant weight.

### 2.5. Characterization

The morphology of the experimental samples was characterized by field-emission scanning electron microscopy (FESEM, JOEL 6700F, Japan, operating at 10 kV).

At the pre-set time points, the soaking specimens were weighed after being cleaned with deionized water and surface-dried, then they were dried to a constant weight in a vacuum at 40 °C and weighed again. The pH value of the PBS was determined by averaging the results of the 3 independent measurements, obtained from the three identical samples for each type of composite. The weight losses were calculated using the following equation:

(1) $$W_{Loss}(\%)=\frac{m_{d}}{m_{0}}\times 100\%$$

where W_Loss_ is the average degrading rate, and m_0_ and m_d_ are the initial and final weights (after removing the surface water), respectively.

The water uptake measurements were calculated using the following equation [18]:

(2) $$W_{water}(\%)=\frac{m_{w}-m_{d}}{m_{d}}\times 100\%$$

where W_water_% is the wateruptake rate, and m_w_, m_o_ and m_d_ are the weight of specimen after conditioning, the final weight (after removing surface water) and the initial weight, respectively.

Gel permeation chromatography (GPC) measurements were taken for the degradation samples in tetrahydrofuran (THF) (analytical purity) at a concentration of 1–2 mg/mL by a Waters 2414 system (Milford, MA) equipped with a Waters Differential Refractometer. THF was eluted at 1.0 mL/min through two Waters Styragel HT columns and a linear column. The internal and column temperatures were kept constant at 35 °C. Calibration curves were obtained based on the standard samples of the mono-dispersed polystyrene.

The thermal analysis of the samples was performed using a differential scanning calorimetry (DSC) instrument (Netzsch Co. Ltd., Freistaat, Germany). The samples, weighing approximately 5–8 mg each, were sealed in an aluminum pan, heated under a nitrogen flow from room temperature to 220 °C at a heating rate of 20 °C/min, isothermally conditioned at 220 °C for 2 min, cooled to 0 °C and then reheated to 220 °C at a heating rate of 10 °C/min. The crystallinity degree (X_c_) of the samples was estimated using the following Equation:

(3) $$\chi_{c}(\%)=\frac{\Delta H_{m}-\Delta H_{cc}}{\Delta H_{m}^{0}}\times 100\%$$

where ΔH_m_ (J/g) is the value of fusion, ΔH_cc_ is the cold crystallization enthalpy obtained during the DSC heating process, ΔH_m_^o^ is the fusion enthalpy of the completely crystalline PLA, and ϕ is the weight fraction of PLA in the sample. The value of PLA is selected as ΔH_m_^0^ = 93.6 J/g [24].

### 2.6. In Vivo Experiment

#### 2.6.1. Animal Models

The in vivo experiments were carried out as described by Reference [24]. Briefly, a total of 10 healthy adult (~1 year) Japanese white rabbits weighing 3 ± 0.2 kg were selected for animal testing, and they were divided in 2 groups, with 5 rabbits in one group, of which 2 rabbits were used as standby samples. One rabbit was implanted with 2 samples, where the left and right legs were implanted with one sample each (ϕ2 × 6 mm). Further, sub-cage feeding was performed for a week and no adverse reactions were found. Rabbits were anesthetized with an intramuscular injection of ketamine (0.2 mL/kg). After that, the hair on the side of the knee in a roughly 5-cm range was shaved. The iodine disinfectant was used to disinfect the knee parts of the rabbit. The anterior lateral patella of the knee was incised to about 4 cm, followed by cutting the skin, lateral support and a joint capsule. A hole with a depth of 1 cm was drilled in the femur and tibial cancellous bone of the knee. The PLA and WPLA rods were implanted into the hole separately; then, postoperative suture, iodophor disinfection and sub-cage feeding were performed. Rabbits were sacrificed with ear veins injected with air. The bone with the implanted rod was removed from the euthanized rabbits after 3, 6, 12 and 18 months, and preserved by a 10% formalin solution. For all the animal experiments, the materials and surgical instruments were radiation-disinfected in the Tianjin Jinpeng far radiation Co., Ltd. after Co60 for 24 h, with a radiation dose of 25 KGy, and the experiments were implemented by Tianjin Hospital (approval No. 2015-11155).

#### 2.6.2. Routine Pathological Examinations

Hard histological biopsies were performed to evaluate the structure variation of the implants under long-term degradation behaviors and the tibial cancellous bone response after surgery. The surgical sites were fixed in a 10% formaldehyde solution, and then the samples were dehydrated in the order of the graded series of alcohols. Following the dehydration and decalcification, the specimens were embedded in paraffin, and the tissue sections were stained with hematoxylin and red staining.

**Figure 1:**
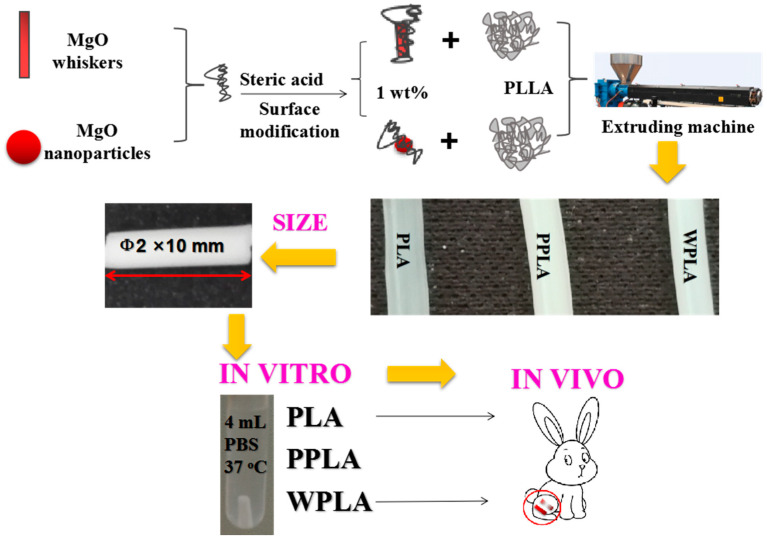
Schematic illustration of the experiment.

The whole process of experiment is present in Figure 1.

## 3. Results

### 3.1. Microstructures of In Vitro Degradation of PLA and Composites

**Figure 2:**
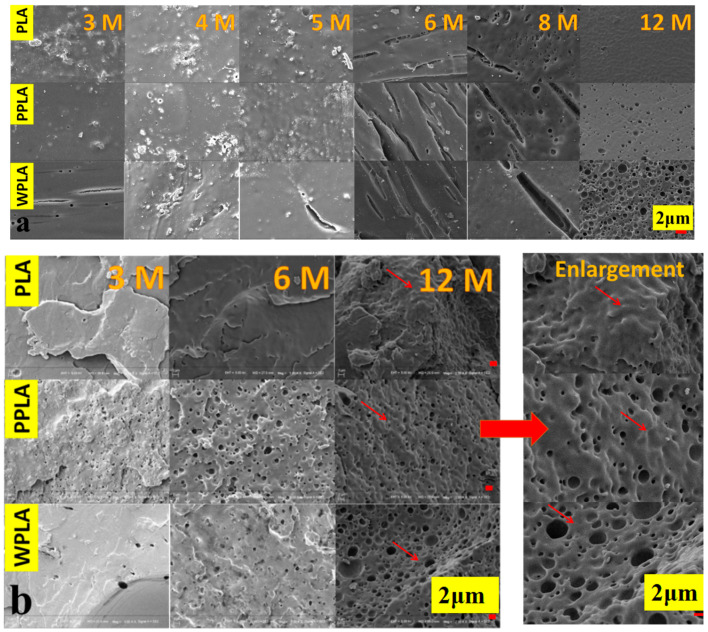
SEM microstructures of polylactic acid (PLA) and composites at different degradation times: (**a**) surface morphologies; (**b**) fracture morphologies.

Figure 2 shows the surface morphologies and fracture morphologies (brittle fractures treated with liquid nitrogen) of the PBS-soaked samples at the different degradation periods. It is obvious that the surface degradation of all specimens is gradually aggravated with the extension of the immersion time. Specifically, some microcracks on the surface of all the samples in the 6 months appear, and the degrading holes are also shown by the 8 and 12 months in Figure 2a, which are produced by the PLA decomposition [25]. Meanwhile, the higher number of decomposing holes of the PPLA and WPLA composites are displayed and indicate that the decomposition of composite materials is more intensive than that of PLA under the long immersion period, probably due to the presence of MgO affecting the matrix’s hydrophilicity. In Figure 2b, the fracture morphologies of the composites also form the greater number of bigger holes of WPLA and PPLA observed in comparison with the contemporary PLA sample and the intensive hydrolysis behavior of the matrix over the 12 months of composition is also displayed, suggesting their accelerating degradation under the long-term immersion. However, it can be observed that the morphologies of the samples in some areas (marked by red arrows) are greatly different between WPLA and the others, particularly in the graphs of 12 months. The former performs brittle-similar fracture behavior, while the latter exhibits noticeable plastification areas, which are probably a result of the degradation product and water [20,21].

### 3.2. Weight Loss, Water Intake and pH Value

**Figure 3:**
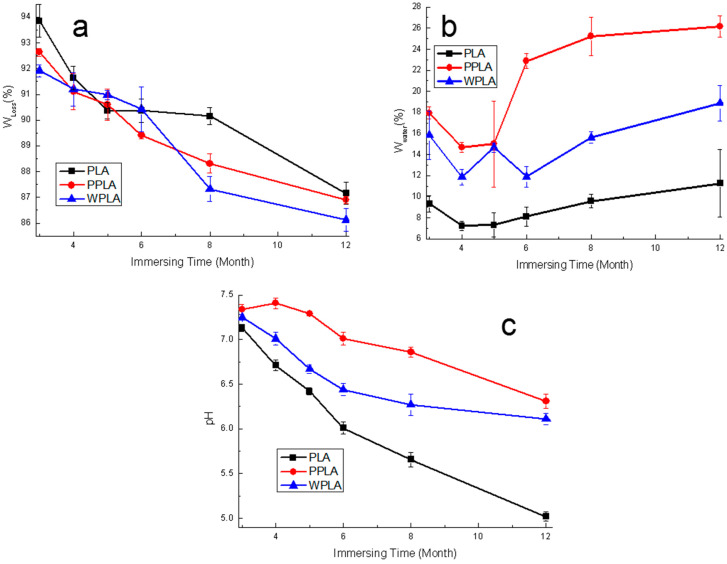
The (**a**) weight loss, (**b**) water intake and (**c**) pH value as a function of the degradation time of the neat PLA and composite immersed in the phosphate buffer solution (PBS).

The weight changes caused by the sample’s degradation in the PBS are shown in Figure 3a. It can be seen that the variation in all samples is similar, namely by decreasing gradually. Obviously, the residual weights of the composites are comparatively less than that of the neat PLA over the 12 months, which is also mainly due to the MgO presence increasing the water uptake and accelerating the matrix decomposition. Initially, from the 4 months, there is an apparent increment in the degrading rate of PLA possibly because of its self-catalytic degradation [26], which accelerates its weight loss. Compared with the control PLA sample, the variation in the composites’ weight loss is not greatly remarkable during the whole testing process. However, it is notable that WPLA provides the slightly more residual weight from the 5 months probably due to the hard degradation of its crystalline part leading to much more water, and there is also the increment in the water intake of WPLA over 5 months. Consequently, PPLA has the higher residual weight in comparison with WPLA in the 12 months. This is probably related to the MgO shape affecting the crystal behaviors and water uptake of the PLA matrix [18]. In Figure 3b, the composites exhibited the higher value of water absorption, which is consistent with their weight loss results, and attributed to MgO enhancing the hydrophilicity of the PLA matrix. Moreover, compared with the contemporary WPLA specimens, the samples of PPLA perform much a stronger hydrophilic capability during the experimental period and this is probably caused by the higher quantity of nanoparticles compared with the same weight whiskers. Undoubtedly, the density of the crystal areas of PPLA can also be increased more notably by nanoparticle nucleation [23] but these forming areas are probably smaller due to the inhibiting growth effect between each other. Moreover, there may be much more parts of the imperfect crystal and amorphous region present, which can easily occur at the beginning of the degradation and lead to the acceleration of the degrading process, since metal oxides can effectively accelerate the decomposition of the PLA matrix, especially in a comparatively short-term degradation [27]. As to the variation in the pH value shown in Figure 3c, the pure PLA has a significantly lower value than that of the composites. With the addition of MgO, the pH values of the degrading solution are higher, in spite of decreasing gradually, especially at the final stage. The delay in the pH reduction in the composite solution may be mainly due to the neutralization of the alkaline magnesium ions released by MgO in the degradation medium [28]. Additionally, it is noted that PPLA also performs slightly better in controlling the solution’s pH value, although the pH value of the later period was around at 6.4.

### 3.3. GPC and DSC Results

In Table 2, the molecular weight data obtained from the GPC analysis are included. It is observed that the *M_w_* of PLA and the composites decrease as the time is prolonged, as a consequence of the hydrolytic process. Initially, there is no intensively significant difference between the composites and PLA. The *M_w_* of WPLA over 3 months is a little higher and this tendency is maintained until the 12-months of the degradation. As to the variation in PI (dispersity indexes), initially, the neat PLA performs the lower value compared with the composites for the samples of 3 and 4 months, while with the time prolonging, there is an increasing tendency of PLA PI. This change is probably due to the hydrolysis of the matrix and the formation of the degradation product with a lower *M_w_* from the amorphous areas in the PLA sample. It is noticeable that the variation in the *M_w_* of PPLA is with the lowest value from 3 months to 6 months, and this is consistent with the previous study’s report that metal oxides can enhance the degradation of the PLA matrix before 3 months [29]. However, with the extension of the immersing time, PPLA has the higher *M_w_* value than that of PLA in 8 and 12 months, indicating the delaying degradation of PPLA. This is probably due to the more crystalline structure formed by the nanoparticles and the slowing degradation of the crystalline part occurring in the polymeric matrix. The MgO, especially the whiskers, is favorable for forming the more crystal regions, resulting in it effectively inhibiting and alleviating the degradation of the specimen [7], in spite of increasing the water uptake and hydrophilicity of the PLA matrix. In addition, there is a prominent difference between the *M_w_* of PPLA and WPLA in the 12 months, and the higher *M_w_* value of WPLA observed is possibly ascribed to the presence of bigger and more complete crystal regions, which lead to a higher crystallinity and is formed by the tighter arrangement of the PLA molecule induced by the whiskers [30]. This also indicates that the large amount of water entering the matrix is apt to accelerate the hydrolyzing of the amorphous material, but it slightly impacts on the crystal area even for the long-term degradation.

**Figure 4:**
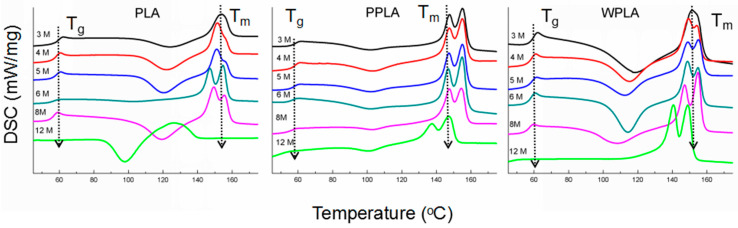
The differential scanning calorimetry (DSC) curves of the secondary heating curves obtained for the degraded samples.

The DSC data for the pure PLA and the composites in the experiment are displayed in Figure 4. During the degradation process, the Tg peaks observed for the samples is gradually shifted to the lower value, and the same variation is also observed for Tm. There are also two endothermic peaks for some curves and this is a common phenomenon for PLA degradation due to the recrystallization process of the defective crystals as decomposed products [7]. The whole degrading process starts with an amorphous degradation and then gradually combines with the destruction of the crystalline structure in a long-term degradation, which is also with the similar decline process of the molecular weight proved by the GPC results. It is found that there is only one endothermic peak in the PLA sample in 12 months with the lower value of Tm, and according to the GPC results in the corresponding period, it probably implies the formation of a decomposed product with a low molecular weight during the long-term degradation, which is distinguished from the crystalline region in PPLA and WPLA, as observed in the contemporary comparison of the curves. For the samples in 3 months, the curve of the PPLA specimen has double the endothermic peaks, possibly due to its accelerating degradation caused by the MgO nanoparticles [29] at the former 3-months stage. Moreover, in comparison with the curves of PPLA, the variation in the WPLA DSC line implies the lower degrading rate of WPLA, and this can be ascribed to its high crystallinity by the whiskers, demonstrated by the results of Xc in Table 3. Additionally, there is also no obvious cold crystallization phenomenon of WPLA in 12 months, differing from the PPLA performance, which also verifies the high crystallization of the WPLA composite. It is also greatly remarkable that the whole variation of PPLA in the DSC from 3 months to 12 months is not similar to that of WPLA, and the double endothermic peaks are maintained in all the curves of PPLA. It is probably due to the relatively denser or more compact crystalline network structure in the PPLA matrix after the short-term degradation, which may be helpful to delay its degradation, in spite of its lower crystallinity.

### 3.4. Dying of In Vivo Degradation

**Figure 5:**
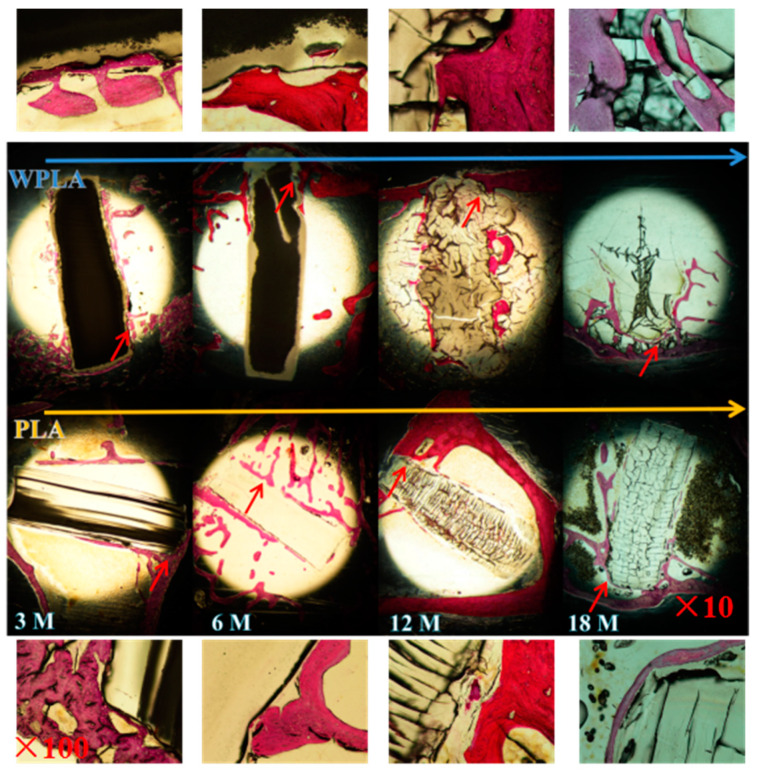
Histological morphologies of the implanted PLA and WPLA after 3-, 6-, 12- and 18-months duration of implantation.

According to the degradation results in vitro, the in vivo degradation of PLA and WPLA were carried out for the comparatively long-term implanting. More attention was given to the changes in the materials in vivo with the time of 3 months, 6 months, 12 months and 18 months, and the graphs of the histological examinations stained by hematoxylin and red staining are displayed in Figure 5. After 3 months, the edge of the implanted WPLA composite was not flat, while the control PLA was still relatively smooth, as shown. Combined with the results of the water intake and molecular weight of the materials, it indicates that the swelling stage of the decomposing process is more intensive for WPLA, due to its enhanced hydrophilicity. Meanwhile, after the 6-month implantation, it is noteworthy that the implants of PLA and WPLA exhibited a large number of decomposition cracks, and the apparent destruction of the implants was also observed (12 months of Figure 5), but the cracks for both samples are not similar. Specifically, there are much more and parallel cracks displayed in the control PLA in spite of the insignificant swelling, nevertheless the specimen of WPLA seems to swell significantly and break into some block regions by less random cracks. The results illustrate that the water intake rate of WPLA with the MgO whiskers is faster than that of the pure PLA, but the degradation rate of the pristine PLA may be more prominent, suggesting that the degradation process of the matrix in the control and composites were different from each other. Moreover, after the 18 months, the implant of WPLA is basically decomposed, the partial degrading matrix enter into the newly-formed bone trabecula with a tight touch (marked by red arrows in 18 months of Figure 5) and PLA is still in the comparatively complete rod shape with a slight expansion, implying the accelerating decomposition of the swelled WPLA in the final degrading stage. This illustrates that MgO has a significant effect on the degradation process of the PLA matrix. The swelling rate and process are more accelerated and more intensively impacted on the degradation, along with the matrix decomposition. Additionally, from the parts noted by the red lines in the graph, it can be seen that the presence of MgO is favorable for inducing the tight connect between the matrix and bone trabeculae, due to both the better hydrophilicity and biological activity of the significant effect of Mg^2+^ on bone formation and healing [28].

## 4. Discussions

The nucleation abilities of the nanoparticles and whiskers for the polymeric composites are different, leading to the variation in their crystallinization. Undoubtedly, with the equal quality, the quantity of the nanoparticles is much more than that of the whiskers, and the hydrophilicity improvements of PPLA are more prominent, as shown in Figure 3b. The hydrolysis process of the composites is mainly impacted by water absorption. The SEM graphs reveal that the degradation behaviors of the composites PPLA and WPLA in the long-term degradation process are similar to that of the pure PLA, and there is the beginning of the amorphous region’s decomposition for the polymeric matrix. However, the decomposition of the PLA amorphous area is obviously faster, which shows the “tough fracture”, although no obvious hole is shown in the 12-months sample of Figure 1. It is probably due to the amorphous status from the degradation product of a low molecular weight [31]. Meanwhile, it also seems that compared with PPLA, the crystalline region of WPLA is more difficult to destroy, possibly due to two aspects. One is that the whiskers in WPLA induce the PLA molecular chain to form a crystal structure along the whiskers, which enhance their interface bonding and increase the crystallinity [30]. The other is that the whiskers have the ability to promote the ordering of the hydrolyzed PLA molecular chains, and its looser percolation network structure is more conducive to the growth of the PLA chip [31], which is also confirmed by the results of a higher value of crystallinity in the DSC and molecular weight in the GPC of the 12-months degradation. Additionally, it should be noted that although PPLA is easier to lose the amorphous area and perform a fast degradation during a short-time immersion, the nanoparticles with a smaller size are apt to fabricate the relatively denser or more compact crystalline structure and this is helpful to delay the PPLA degradation in the long-term degradation, which can also maintain the pH stability, as shown in Figure 2.

As to the in vivo degradation, the variation in the decomposing process of the WPLA composite affected by the whiskers is also distinct from that of the control PLA. The long-term degradation results show that the composites swell more prominently and faster due to the enhanced hydrophilicity which increases the water intake, and the swelling rate of the composite is also much faster than the degradation rate of the polymeric matrix. Meanwhile, the degraded status of the WPLA implant observed directly from the histological morphologies differs from the sample without whiskers. Due to the swelling effect, the composite is decomposed into the “block” shape, although it still degraded through bulk erosion [32], which is identical to the degrading mechanism of the pristine PLA. However, with the higher water uptake, it indicates that the degradation of WPLA is greatly controlled by the diffusion rate of the water in the PLA matrix due to the significant enhancement of hydrophilicity. Moreover, the biological activity of the PLA matrix is obviously improved, the specimen is tightly surrounded by bone tissue and the materials and newly formed bone trabecula are integrated into each other.

## 5. Conclusions

The in vitro and in vivo degradation of the biodegradable composites prepared by the PLA matrix blending with the 1% wt. MgO were investigated to determine the effect of the MgO shape (nanoparticles and whiskers) on the degradation process of PLA/MgO composites. We have demonstrated that the addition of MgO can accelerate the water uptake rate of the PLA matrix, and the degrading procedure of composites begins with the loss of the non-crystalline region prior to the destruction of the crystalline parts, demonstrated by the DSC results, but the water uptake rate of the composites, especially with MgO whiskers, is obviously faster than its bulk degradation in the degrading procedure, caused by the presence of MgO. The in vivo results of the histological morphologies suggest that the PLA matrix of the composite has the prominently enhanced bioactivity and that this PLA/MgO biocomposite can be potentially utilized for bone repair with better performance.

## Figures and Tables

**Table 1:** Detailed information of the samples.

Sample	SpMgO Content/PLA Content (w/w)	SwMgO Content/PLA Content (w/w)
PLA	0	0
PPLA	1/100	0
WPLA	0	1/100

**Table 2:** Gel permeation chromatography (GPC) data corresponding to the samples.

Time/Months	PLA			PPLA			WPLA		
	*M_w_*	*M_n_*	PI	*M_w_*	*M_n_*	PI	*M_w_*	*M_n_*	PI
3	135,777	85,630	1.59	124,969	79,492	1.57	142,721	87,717	1.63
4	122,638	78,164	1.57	119,200	74,087	1.61	128,695	78,720	1.63
5	113,343	70,064	1.62	105,310	65,020	1.62	111,342	75,066	1.48
6	84,728	55,543	1.53	77,485	50,884	1.52	103,873	67,122	1.55
8	51,364	28,036	1.83	64,290	41,053	1.57	68,602	44,645	1.54
12	4017	3365	1.22	9798	16,703	1.70	21,573	13,011	1.66

Weight averaged molecular weight (*M_w_*), number averaged molecular weight (*M_n_*) and dispersity indexes (PI = *M_w_*/*M_n_*) of the specimens as determined by GPC.

**Table 3:** DSC data corresponding to the samples.

Time(Months)/Samples		*T_m1_* (°C)	*T_m2_* (°C)	Δ*H_cc_* (J/g)	Δ*H_m_* (J/g)	*X_c_* (%)
3	PLA	153.9	-	−12.8	25.5	40.8
	PPLA	147.7	155.2	−8.37	35.3	46.6
	WPLA	151.7	154.5	−23.6	37.7	65.4
4	PLA	151.5	156.6	−21.0	33.1	57.8
	PPLA	147.6	155.2	−10.9	37.5	51.6
	WPLA	149.4	154.2	−22.1	34.3	60.2
5	PLA	151.4	156.8	−25.0	29.6	58.4
	PPLA	147.3	155.1	−6.3	38.9	48.4
	WPLA	149.1	155.1	−22.1	30.6	56.2
6	PLA	147.2	154.7	−5.0	39.6	47.6
	PPLA	146.8	154.9	−5.9	43.0	52.2
	WPLA	148.8	154.8	−30.3	32.9	67.5
8	PLA	149.4	155.6	−26.5	45.6	77.1
	PPLA	147.7	154.6	−4.8	35.8	43.3
	WPLA	147.1	154.9	−16.8	43.0	63.8
12	PLA	126.9	-	−33.0	24.9	61.9
	PPLA	137.5	147.2	−6.8	28.7	38.0
	WPLA	140.6	148.9	−4.0	43.1	50.3

## Supplementary Materials

The following are available online at https://www.mdpi.com/2073-4360/12/5/1074/s1. Figure S1. The SEM graphs of the MgO nanoparticles and whiskers.
