# Supplementary figures and images for: Effects of Magnesium Oxide (MgO) Shapes on In Vitro and In Vivo Degradation Behaviors of PLA/MgO Composites in Long Term
Source: Polymers (Basel). 2020 May 8;12(5):1074. doi: 10.3390/polym12051074 (PMC7284841; doi:10.3390/polym12051074)

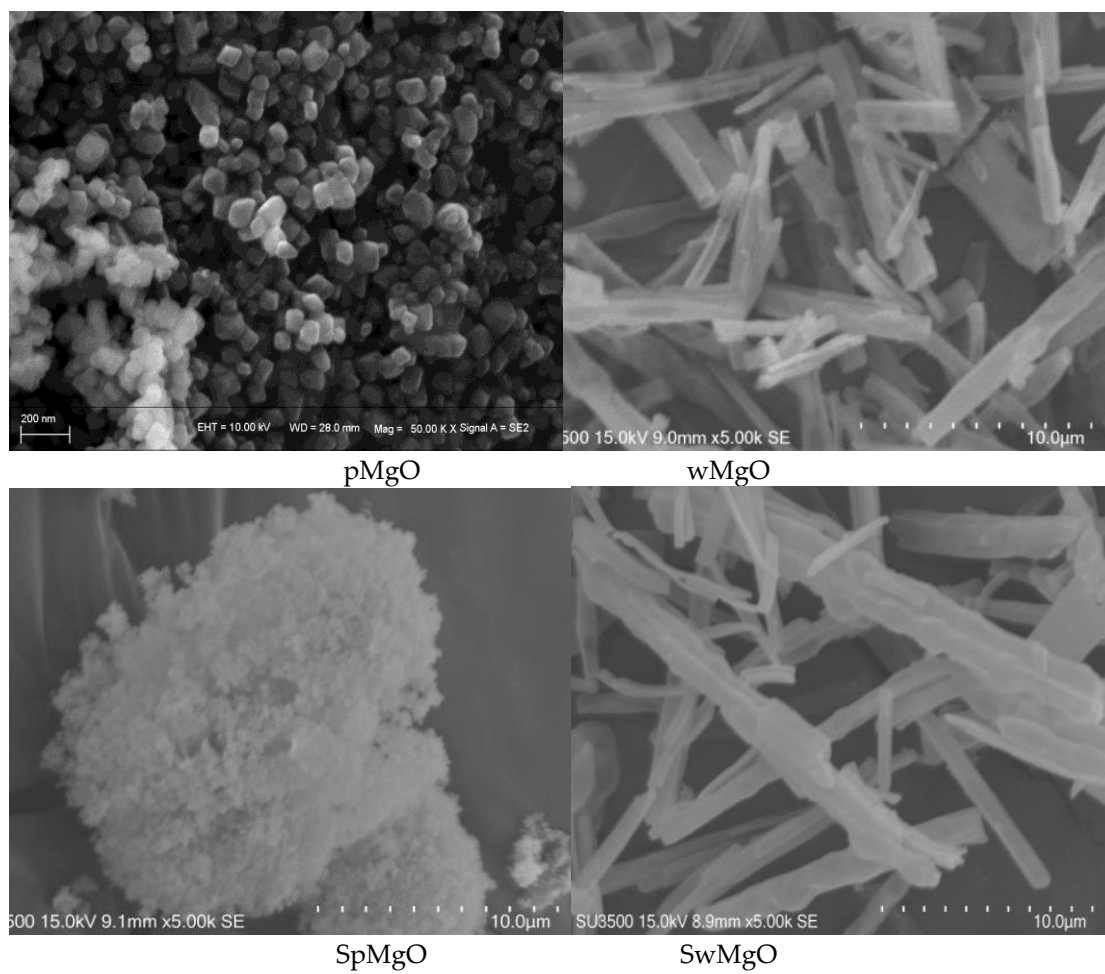

**Figure 1.** The SEM graphs of pMgO, wMgO, steaic acid modified pMgO and wMgO.

Supplement: Supplementary file 1 [file polymers-12-01074-s001.pdf]
